# Depressive and Anxiety Symptoms Are Predictors of Seizure Recurrence in Adults With Newly Diagnosed Epilepsy

**DOI:** 10.3389/fpsyt.2021.784737

**Published:** 2021-11-24

**Authors:** Rui Zhong, Qingling Chen, Xinyue Zhang, Nan Li, Weihong Lin

**Affiliations:** ^1^Department of Neurology, First Hospital, Jilin University, Changchun, China; ^2^Department of Hepatology, Tianjin Medical University, Tianjin Second People's Hospital, Tianjin, China

**Keywords:** depressive symptoms, anxiety symptoms, newly diagnosed epilepsy, seizure recurrence, predictor

## Abstract

**Objective:** To investigate whether emerging depressive and anxiety symptoms are predictors of seizure recurrence in a cohort of patients with newly diagnosed epilepsy (PWNDE) who did not have a history of psychiatric diagnosis.

**Methods:** A cohort of 283 PWNDE were psychiatrically assessed before antiseizure medication (ASM) therapy and were followed for 12 months to assess seizure recurrence. The influence of depressive and anxiety symptoms score on seizure recurrence was assessed using univariate and multivariate binary logistic regression analysis. Receiver operating characteristic (ROC) curve analysis was utilized.

**Results:** A total of 283 individuals were included in final analysis, and 115 patients (40.6%) experienced seizure recurrence during follow-up. In multivariate logistic regression analysis, NDDI-E and GAD-7 score were associated with an increased risk of seizure recurrence with an adjusted OR of 1.360 (CI: 1.176–1.572; *P* < 0.001) and 1.101 (CI: 1.004–1.209; *P* = 0.041), respectively. Additionally, the adjusted OR and 95% CI of seizure recurrence for the “high NDDI-E score and high GAD-7 score” vs. “not high NDDI-E score and not high GAD-7 score” was 7.059 (3.521–14.149) (*P* for trend < 0.001).

**Conclusion:** We found that an emergence of new psychiatric symptoms including depressive and anxiety symptoms were predictors of seizure recurrence in adults with newly diagnosed epilepsy who did not have psychiatric history.

## Introduction

Epilepsy is one of the most common severe brain diseases, affecting more than 70 million subjects worldwide (1). About 30–69% patients with newly diagnosed epilepsy (PWNDE) had further seizures even starting appropriate ASM therapy (2, 3). Physicians would benefit from information regarding which PWNDE may be at higher risk for seizure recurrence after taking ASM therapy. Multiple prior investigations focused on the useful predictors of seizure recurrence such as imaging (4), EEG (5), and epilepsy-related variables (6) in newly treated patients with epilepsy (PWE).

Epilepsy has been always related with psychiatric disorders. The role of psychiatric disorders in epilepsy risk and seizure outcomes had been described previously (7). Psychiatric disorders included depression and anxiety could be predicted by stressful life events (8). A cohort study provided evidence that individuals who had lost a child had an increased risk of being diagnosed with epilepsy, indicating the relationship between risk of epilepsy, psychiatric symptoms and stress (7). Lifetime mood disorder and lifetime generalized anxiety disorder were shown to increase the risk for seizure recurrence in adults with a single unprovoked seizure or newly diagnosed epilepsy (3). In a cohort of patients with epilepsy (PWE) who received temporal lobe resection to treat epilepsy, a psychiatric lifetime diagnosis was associated with lower seizure-freedom rates and worse surgical outcomes (9). Thus, there may exist more complex cerebral pathologies in PWE with psychiatric history.

To date, however, the literature is sparse on the relationship between an emergence of new psychiatric symptoms at epilepsy diagnosis and seizure recurrence in PWE without psychiatric history. Thus, we investigated whether emerging depressive and anxiety symptoms are associated with seizure recurrence in a cohort of PWNDE without a history of psychiatric diagnosis.

## Methods

### Study Sample

This study cohort consisted of 283 adult (≥18 years old) patients from the epilepsy center of Jilin University, First Hospital between June 2016 and May 2020. We included PWNDE who completed the depressive and anxiety symptoms assessments before taking ASM treatment, and all participants had a follow-up period of 12 months after diagnosis. Epilepsy was defined according to International League Against Epilepsy criteria (defined as: two unprovoked seizures occurring more than 24 h apart; a single unprovoked seizure if recurrence risk is high [i.e., >60% over the next 10 years]; or a diagnosis of an epilepsy syndrome) (1, 10). We excluded subjects who (1) were treated with an ASM previously; (2) had a history of psychiatric disorders (lifelong anxiety and depression); (3) had a history of non-epileptic seizures, severe brain diseases other than epilepsy (e.g., dementia or Parkinson's disease), serious physical diseases (e.g., significant hepatic, renal, or cardiopulmonary conditions). Individuals without the physical, mental, and language ability to complete the self-reported questionnaires and interview were also excluded. Total participants provided written informed consent, and the study was approved by the ethics committee of our hospital.

### Assessment of Psychiatric Symptoms

Psychiatric symptoms were assessed at enrollment using the Neurological Disorders Depression Inventory for Epilepsy (NDDI-E) (11) and the Generalized Anxiety Disorder-7 questionnaire (GAD-7) (12), respectively. The NDDI-E is a rapid and user-friendly clinical instrument to screen for the severity of depressive symptoms in PWE (11). The severity of anxiety symptoms was measured with the 7-item GAD-7 (12). We have used the validated versions for the Chinese population (13, 14). A higher scores indicating higher levels of psychiatric symptoms.

### Assessment of Other Potential Predictors

Other potential predictors of seizure recurrence used for data analysis were collected and recorded from the face to face interview at baseline. These potential predictors consisted of age, gender, seizure number before ASM treatment, epilepsy type, presence of a lesion on magnetic resonance imaging (MRI), frequent epileptiform discharges in EEG, and family history of seizures. The pretreatment seizure number was stratified into patients who had ≤5 seizures before starting an ASM, and patients who experienced >5 seizures. The epilepsy type were categorized as focal epilepsy, generalized epilepsy or unclassified epilepsy. The presence of a lesion was determined using preoperative MRI scans. A 24 h video-EEG monitoring study (V-EEG) was performed for each patients to identify the presence of epileptiform discharges (focal or generalized spike-waves). All MRI and EEG reports were made by clinicians with fellowship training. Their reports were subsequently reviewed by a second epileptologist, who made a final classification for this study. We defined a family history of unprovoked seizures as seizures occurring in a first-degree relative (parent or sibling).

### Follow-Up Seizure Recurrence Assessments

All participants were reassessed after their epilepsy diagnosis, and the primary outcome was seizure recurrence during the 12 months follow-up period. Formal follow-up outcome measurements were undertaken at 3, 6, and 12 months after diagnosis. Patients and their proxies were questioned about seizure recurrence at each time by the phone. Data on medication compliance and changes, and potential adverse effects were also recorded. A recurrent seizure was defined as the first unprovoked seizure occurring >24 h after the epilepsy diagnosis.

### Statistical Analysis

Results were expressed as percentages for categorical variables and median (interquartile ranges, IQRs) for the continuous variables. Proportions were compared using the Chisquare test, and student's *t*-tests were employed for the normally distributed variables, while the Mann–Whitney U-test was employed for the asymmetrically distributed variables. The influence of depressive and anxiety symptoms score on seizure recurrence was assessed using univariate and multivariate binary logistic regression analysis with significant confounding factors tested in the univariate analysis adjusted. Results were expressed as adjusted odds ratios (OR) with the corresponding 95% confidence intervals (CI). For a more detailed exploration of the NDDI-E/GAD-7 score-seizure recurrence relationships, the relationship between cross classification of NDDI-E and GAD-7 score and seizure recurrence was assessed using univariate and multivariate logistic regression. Adjusted OR and 95% CI for seizure recurrence was calculated for the “high NDDI-E score and high GAD-7 score” (most risky group) with “not high NDDI-E score and not high GAD-7 score” (least risky group) as the reference. We defined NDDI-E ≥ median as high NDDI-E score. Similarly, GAD-7 score were categorized into high (GAD-7 ≥ median) and not high (GAD-7 < median) with equal sample sizes. Equal sample size in each subgroup of the total sample size gives more power of the test than unequal sample sizes (10). Receiver operating characteristic (ROC) curve analysis was utilized to evaluate the cut-off values on the NDDI-E and GAD-7 score at enrollment with the greatest sensitivity and specificity to predict seizure recurrence in PWNDE. The area under the curve (AUC) was calculated to test the overall prognostic accuracy of NDDI-E score and GAD-7 score. All data were analyzed with SPSS for Windows, version 26.0 (SPSS Inc., Chicago, IL, USA). The two-sided *P* < 0.05 were considered statistically significant.

## Results

Among the 317 individuals identified, 283 completed follow-up assessments and included in the final analysis. Twenty four subjects were excluded for one of the following reasons: disagreed to participant (*n* = 7), treated with ASM previously (*n* = 5), withdraw (*n* = 4), never started or ceased ASM treatment (*n* = 10), lost follow-up (*n* = 8). Table 1 shows detailed demographic and clinical characteristics of total participants. The study subjects had a median age of 35.00 years and a male percentage of 59.7%. In this cohort, 115 patients (40.6%) experienced seizure recurrence during 12 months follow-up period. The median NDDI-E and GAD-7 score were 8 and 5, respectively.

**Table 1:** Clinical characteristics and univariate comparisons for seizure recurrence in PWNDE.

**Variable**	**Seizure recurrence group** **(*n =* 115)**	**No seizure recurrence group** **(=168)**	***P*-value**	**All cases** **(*n =* 283)**
Age (Years), median (IQR)	35 (23, 48)	34 (23, 54)	0.751	35 (23, 51)
Gender				
Male	67 (58.3%)	102 (60.7%)	0.679	169 (59.7)
Female	48 (41.7%)	66 (39.3%)		114 (40.3)
>5 seizures pretreatment				
Yes	78 (67.8%)	57 (33.9%)	<0.001	135 (47.7)
No	37 (32.2%)	111 (66.1%)		148 (52.3)
Epilepsy type				
Focal	92 (80.0%)	125 (74.4%)	0.549	217 (76.7)
Generalized	15 (13.0%)	27 (16.1%)		42 (14.8)
Unclassified	8 (7.0%)	16 (9.5%)		24 (8.5)
MRI lesion				
Yes	59 (51.3%)	44 (26.2%)	<0.001	103 (36.4)
No	56 (48.7%)	124 (73.8%)		180 (63.6)
EEG frequent epileptiform discharges				
Yes	74 (64.3%)	62 (36.9%)	<0.001	136 (48.1)
No	41 (35.7%)	106 (63.1%)		168 (59.4)
Family history of seizures				
Yes	11 (9.6%)	11 (6.5%)	0.352	22 (7.8)
No	104 (90.4%)	157 (93.5%)		261 (92.2)
NDDI-E score, median (IQR)	11 (7, 13)	7 (6, 9)	<0.001	8 (6, 11)
GAD-7 score, median (IQR)	7 (4, 12)	3 (1, 6)	<0.001	5 (2, 8)

*PWNDE, patients with newly diagnosed epilepsy; IQR, interquartile range; NDDI-E, Neurological Disorders Depression Inventory for Epilepsy; GAD-7, Generalized Anxiety Disorder-7*.

The results indicated that patients in seizure recurrence group were more likely to have > 5 seizures before ASM treatment (*p* < 0.001), have a structural lesion in MRI (*p* < 0.001), and have frequent epileptiform discharges in EEG (*p* < 0.001) compared with those in non-seizure recurrence group. There was also a significant inter-group difference in NDDI-E (*p* < 0.001) and GAD-7 score (*p* < 0.001). Patients who had seizure recurrence reported higher depressive and anxiety symptoms levels. No association was found between age, gender, epilepsy type, or family history of seizures and the presence of seizure recurrence. For details see Table 1.

In univariate logistic regression analysis, >5 seizures pretreatment (OR = 4.105; 95% CI: 2.477–6.803; *P* < 0.001), a structural lesion in MRI (OR = 2.969; 95% CI: 1.797–4.905; *P* < 0.001), and frequent epileptiform discharges in EEG (OR = 3.086; 95% CI: 1.883–5.056; *P* < 0.001) were associated with an increased odds ratio for seizure recurrence in individuals with newly diagnosed epilepsy. NDDI-E and GAD-7 scores were associated with an increased risk of seizure recurrence with an unadjusted OR of 1.499 (CI: 1.349–1.667; *P* < 0.001) and 1.241 (CI: 1.163–1.324; *P* < 0.001), respectively. In multivariate logistic regression analysis, NDDI-E and GAD-7 score were still associated with an increased risk of seizure recurrence with an adjusted OR of 1.360 (CI: 1.176–1.572; *P* < 0.001) and 1.101 (CI: 1.004–1.209; *P* = 0.041), respectively, after adjustment for above recorded variables including >5 seizures pretreatment, MRI lesion, and EEG frequent epileptiform discharges. For details see Table 2.

**Table 2:** Univariate and multivariate logistic regression analysis for the seizure recurrence in PWNDE.

**Variable**	**Univariate analysis**		**Multivariate analysis**	
	**Unadjusted OR (95% CI)**	***P*-value**	**Adjusted OR (95% CI)**	***P*-value**
Age (Years) (increase per unit)	0.996 (0.982–1.010)	0.586	–	–
Gender	0.903 (0.557–1.464)	0.903	–	–
>5 seizures pretreatment	4.105 (2.477–6.803)	<0.001	4.144 (2.203, 7.793)	<0.001
Epilepsy type	0.980 (0.597–1.609)	0.937	–	–
MRI lesion	2.969 (1.797–4.905)	<0.001	3.310 (1.726, 6.350)	<0.001
EEG frequent epileptiform discharges	3.086 (1.883–5.056)	<0.001	2.288 (1.216, 4.307)	0.01
Family history of seizures	1.510 (0.631–3.609)	0.354	-	-
NDDI-E score (increase per unit)	1.499 (1.349–1.667)	<0.001	1.360 (1.176, 1.572)	<0.001
GAD-7 score (increase per unit)	1.241 (1.163–1.324)	<0.001	1.101 (1.004, 1.209)	0.041

*PWNDE, patients with newly diagnosed epilepsy; OR, odds ratio; CI, confidence interval; NDDI-E, Neurological Disorders Depression Inventory for Epilepsy; GAD-7, Generalized Anxiety Disorder-7*.

Total patients were divided into four groups according to NDDI-E score and GAD-7 score (Shown in Table 3). Distribution of individuals with seizure recurrence across the NDDI-E and GAD-7 ranged between 19.8% (not high NDDI-E score [NDDI-E < median] and not high GAD-7 score [GAD-7 < median]) to 62.3% (high NDDI-E score [NDDI-E ≥ median] and high GAD-7 score [GAD-7 ≥ median]). The univariate and multivariate logistic regression analysis between the cross classification of NDDI-E and GAD-7 score and seizure recurrence is described in Table 3. Patients with the “high NDDI-E score and high GAD-7 score” had a significantly higher risk of seizure recurrence compared with those with “not high NDDI-E score and not high GAD-7 score.” In multivariate logistic regression model, the adjusted OR and 95% CI of seizure recurrence for the “high NDDI-E score and high GAD-7 score” vs. “not high NDDI-E score and not high GAD-7 score” was 7.059 (3.521–14.149) (*P* for trend < 0.001).

**Table 3:** OR and 95% CI for seizure recurrence according to the cross classification of NDDI-E and GAD-7 scores in PWNDE.

	**GAD-7 score** **+** **NDDI-E score**				
	**GAD-7 < median +** **NDDI-E < median**	**GAD-7 ≥ median NDDI-E < median**	**GAD-7 < median +** **NDDI-E ≥ median**	**GAD-7 > median +** **NDDI-E ≥ median**	***P*-value**
n with seizure recurrence/All cases	21/106	10//29	13/34	71/114	
Univariate model	1.00 (reference)	2.130 (0.864–5.253)	2.506 (1.081–5.807)	6.683 (3.633–12.295)	<0.001
Multivariate model^a^	1.00 (reference)	2.709 (0.990–7.414)	1.656 (0.647–4.235)	7.059 (3.521–14.149)	<0.001

a *All multivariate models adjusted for >5 seizures pretreatment, MRI lesion, and EEG frequent epileptiform discharges*.

*PWNDE, patients with newly diagnosed epilepsy; OR, odds ratio; CI, confidence interval; NDDI-E, Neurological Disorders Depression Inventory for Epilepsy; GAD-7, Generalized Anxiety Disorder-7*.

**Figure 1:**
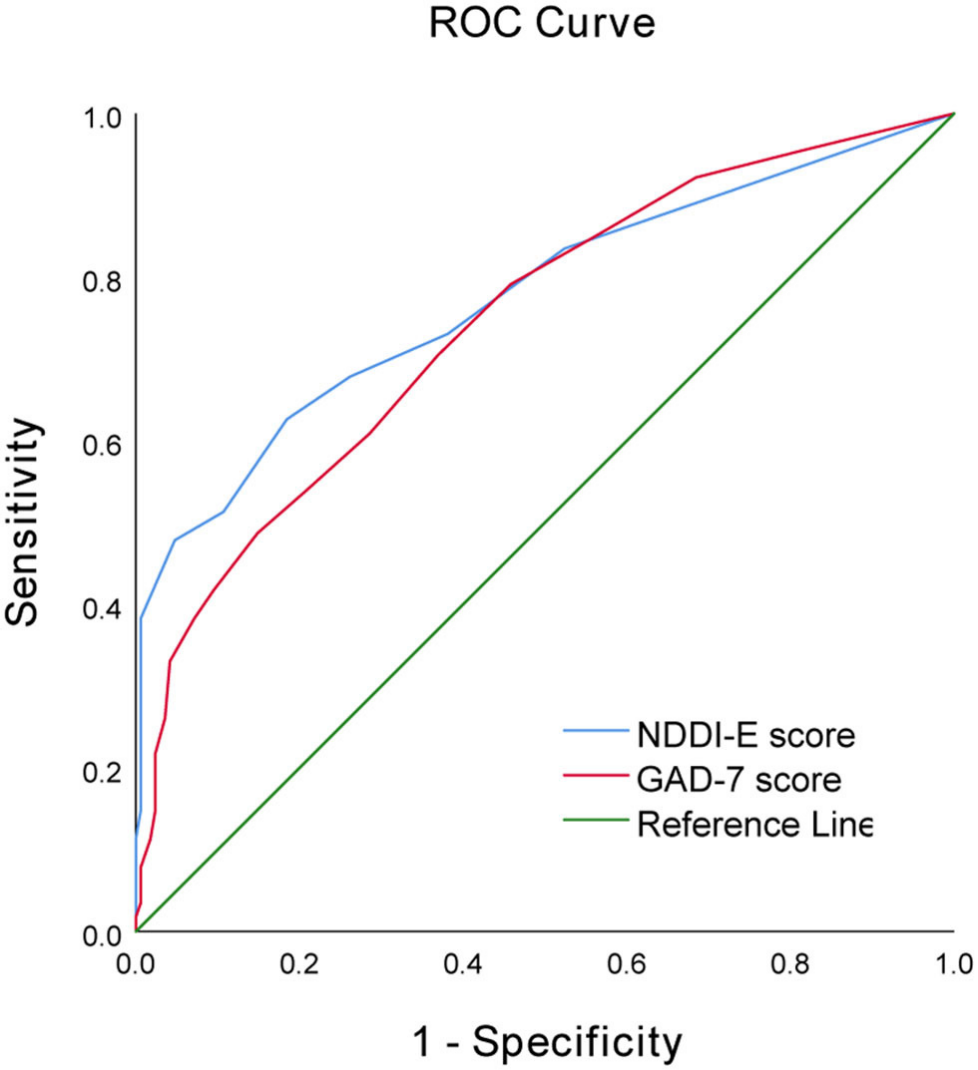
Receiver operator characteristic curve demonstrating sensitivity as a function of 1-specificity for predicting seizure recurrence based on the NDDI-E and GAD-7 scores in PWNDE.

Using ROC curve, NDDI-E score ≥9 at epilepsy diagnosis predicts the seizure recurrence during 12 months follow-up period, with a sensitivity of 67.8% and a specificity of 73.8%; area under the curve (AUC) = 0.775, 95% CI: 0.717–0.833; *P* < 0.001). Additionally, the optimal cut-off point of GAD-7 score as an indicator for prediction of seizure recurrence was projected to be 5, which yielded a sensitivity of 70.4% and a specificity of 63.1%, with the area under the curve (AUC) at 0.744 (95% CI: 0.686–0.803; *p* < 0.001). For details see Table 4 and Figure 1.

**Table 4:** Receiver operating characteristics curve analysis of seizure recurrence according to NDDI-E and GAD-7 scores.

**Test result value**	**AUC**	**Std. error**	**Asymptotic sig**.	**95% CI**		**Cut-off value**	**Sensitivity**	**Specificity**
				**Lower bound**	**Upper bound**			
NDDI-E score	0.775	0.3	<0.001	0.717	0.833	9	0.678	0.738
GAD-7 score	0.744	0.3	<0.001	0.686	0.803	5	0.704	0.631

*AUC, area under the curve; CI, confidence interval; NDDI-E, Neurological Disorders Depression Inventory for Epilepsy; GAD-7, Generalized Anxiety Disorder-7*.

## Discussion

To our knowledge, it is the first study aimed to identify whether the emerging depressive and anxiety symptoms are associated with seizure recurrence in a cohort of individuals with newly diagnosed epilepsy who did not have psychiatric history. Our findings demonstrated that both depressive and anxiety symptoms are significant predictors of seizure recurrence in patients during the first 12 months of ASM therapy. This study replicates the findings of a prior research indicating that greater neuropsychiatric symptomatology is a independent predictor of failure to achieve seizure control in newly treated patients with epilepsy (15).

Our data illustrated that pretreatment depressive and anxiety symptoms were power predictors of high risk of experiencing seizure recurrence after epilepsy diagnosis. Depressive and anxiety symptoms scores had similar diagnostic ability to predict the further seizure in PWNDE. The patient group with high scores of both depressive and anxiety symptoms (≥median) was associated with a 7.058-fold increase in risk of seizure recurrence compared with patients with not high scores of both psychiatric symptoms (<median). Consistent with our findings, Petrovski et al. reported that greater neuropsychiatric symptomatology is predictive of the failure of seizure control in PWNDE newly treated with ASM (15). In their study, neuropsychiatric symptomatology was assessed using the A-B Neuropsychological Assessment Scale (ABNAS) which is a validated brief scale of cognitive and behavioral function (16). The 24-item ABNAS questionnaire was developed specifically to assess patient-perceived cognitive effects of ASM therapy and has been illustrated to correlate with scales for memory, and psychiatric levels (17). In our study, the NDDI-E and GAD-7 are rapid and user-friendly clinical instruments to screen for the severity of depressive and anxiety symptoms, respectively, which are validated brief scales for Chinese adults with epilepsy (13, 14). These two scales could be completed by a patient in about 2–3 min, which make it a practical tool for routine clinical use. Additionally, there may exist a relationship between psychiatric symptoms and the frequency of pre-treatment seizures in PWNDE. However, the results of multivariate analyses suggested that this potential relationship did not influence the predictive role of psychiatric symptoms in recurrent seizure in patients.

The significant role that psychiatric history may play in provoking seizures (7) and aggravating pharmacoresistant seizures (18, 19) has been increasingly recognized. Lifetime mood disorder and lifetime generalized anxiety disorder were reported to be associated with an increased risk for seizure recurrence in subjects with a single unprovoked seizure or newly diagnosed epilepsy (7). A lifetime history of depression was shown to increase the risk for intractable epilepsy in newly diagnosed and treated individuals with epilepsy (19). Similar associations were also reported in patients who received temporal lobe resection to treat epilepsy (9). Multiple prior studies confirmed that a history of psychiatric disorders is a predictor of the failure to achieve seizure freedom after temporal lobe epilepsy surgery and worse postoperative seizure outcome (9, 18, 20). However, it remain unclear whether an emergence of new psychiatric symptoms provides prognostic information regarding success of seizure control in newly diagnosed and treated individuals with epilepsy. Our findings in this study showed that emerging psychiatric symptoms including depressive and anxiety symptoms were associated with an increased risk of seizure recurrence in PWNDE who did not have psychiatric history. A recent study reported that depressive symptoms were more severe in patients with higher seizure frequency during the COVID-19 pandemic (21). Seizure during pandemic, and altered use of ASM were found to be associated with depression during COVID-19 pandemic (22). Combined with the existing evidence, psychiatric symptoms at epilepsy diagnosis and psychiatric history were both predictive of the failure of seizure control in PWNDE even starting appropriate ASM therapy.

One possible explanation for our findings is that brain network changes in psychiatric disorders could have an impact on the course of epilepsy. Neuroimaging studies showed the prominent role of deficits of brain networks in psychiatric disorders, including the prefrontal cortex, amygdala, and insula (23). Lee et al. observed the disruption of brain network in mesial temporal lobe epilepsy (24). Evidence also showed the alterations of the white matter network and structural connectivity in non-lesional temporal lobe epilepsy (25). Psychiatric disorders involve network-level changes and network dysfunction, and network dysfunction is commonly manifested as seizures in epilepsy (26). Additionally, a hyperactivity of the hypothalamic—pituitary—adrenal (HPA) axis was associated with cortical changes especially in the volume of hippocampus and frontal lobes, which not only lead to depression but also contribute to epilepsy (27, 28). Excess hormone release caused by stress or depression may also exacerbate epileptogenesis (29).

Several limitations should be acknowledged in this study. First, follow-up data on seizure recurrence, medication compliance and changes were gathered based on self-report. Seizures occurring during sleeping and complex partial seizure are always not documented because the patient may be less aware of them (30). Thus, there may exist self-report bias. Second, we do not have reliable information on counseling or psychological treatments. This variable was not included was a possible confounder, and its potential effects on seizure outcome was not assessed. Third, our cohort comprised adults of a wide range of ages. Psychiatric symptoms may have different impacts on seizure recurrence, depending on the age group.

In conclusion, we found that an emergence of new psychiatric symptoms including depressive and anxiety symptoms at the time of diagnosis were associated with an increased risk of seizure recurrence in adults with newly diagnosed epilepsy who did not have psychiatric history. Our findings might instigate prospective studies to investigate whether the treatment of psychiatric symptoms reduce risk of seizure recurrence in newly diagnosed and treated patients with epilepsy.

## Data Availability Statement

The raw data supporting the conclusions of this article will be made available by the authors, without undue reservation.

## Ethics Statement

The studies involving human participants were reviewed and approved by the Ethics Committee of the First Hospital of Jilin University. The patients/participants provided their written informed consent to participate in this study.

## Author Contributions

WL and RZ conceived and designed the study. RZ, XZ, and NL were involved in data acquisition. QC and RZ analyzed the data and wrote the manuscript. All authors contributed to the article and approved the submitted version.

## Funding

This work was supported by a grant from the Program of Jilin University First Hospital Clinical Cultivation Fund (LCPYJJ2017006).

## Conflict of Interest

The authors declare that the research was conducted in the absence of any commercial or financial relationships that could be construed as a potential conflict of interest.

## Publisher's Note

All claims expressed in this article are solely those of the authors and do not necessarily represent those of their affiliated organizations, or those of the publisher, the editors and the reviewers. Any product that may be evaluated in this article, or claim that may be made by its manufacturer, is not guaranteed or endorsed by the publisher.
